# Thermodynamic Studies and Optimization of the Method for Obtaining Neodymium Fluoride for the Production of Magnetic Sensors’ Sensitive Elements

**DOI:** 10.3390/s21248361

**Published:** 2021-12-15

**Authors:** Andrei N. Kropachev, Sergey V. Podrezov, Alexander V. Aleksakhin, Andrey A. Gudilin, Olga A. Kondratyeva, Lyudmila N. Korshunova

**Affiliations:** 1 Department of Non-ferrous Metals and Gold, College of Ecotechnologies and Engineering, National University of Science and Technology “MISiS”, 119991 Moscow, Russia; 2 Department of Mining Equipment, Transport and Mechanical Engineering, College of Mining, National University of Science and Technology “MISiS”, 119991 Moscow, Russia; aleksahin.av@misis.ru (A.V.A.); 27-01-1982@mail.ru (A.A.G.); kondratyeva.oa@misis.ru (O.A.K.); lnkorshunova76@gmail.com (L.N.K.)

**Keywords:** thermodynamics, rare earth metals, ammonium hydrofluoride, fluorination, heating stepwise

## Abstract

Rare earth metals (REM) with magnetic properties find application in the recently developed high-tech industries. Sensor magnetic systems based on neodymium are increasingly in demand in modern engineering and geological surveys due to their favorable combination of properties of magnetic materials based on rare earth metals. One of the problems is to obtain high-quality materials for the production of such magnetic sensors. It should be noted that the high activity of REM does not allow obtaining master alloys and REM-based alloys from metallic materials; it is advisable to use halide compounds. This work discusses a method for producing neodymium fluoride from its oxide. REM fluorides can be obtained by fluorinating the oxides of these metals. Various fluorine-containing compounds or elemental fluorine are usually used as fluorinating reagents, which have their own advantages and disadvantages. The thermodynamic and technological analysis of neodymium fluoride production processes has shown the most acceptable fluorinating agent is ammonium hydrofluoride, which was used in this work. In order to increase the productivity and degree of chemical transformation, it was proposed to perform heating stepwise; i.e., at the initial stage, heat at a speed of 3 degrees per minute, after which the heating speed was reduced to 2 degrees per minute, and finally the speed was reduced to 1 degree per minute. Due to proposed heating mode, the same productivity and yield of chemical transformation were achieved, with an increased efficiency up to 30%, which can significantly reduce the cost of production. The obtained product is used in the production of neodymium-based alloys by metallothermic reduction of a mixture of fluorides. The sensor material obtained in this way is characterized by a low (less than 0.05%) oxygen content.

## 1. Introduction

Sensor magnetic systems based on neodymium, dysprosium and terbium are increasingly in demand in modern engineering and geological surveys due to their favorable combination of properties of magnetic materials based on rare earth metals, which was demonstrated in very recent research. New studies are conducted in the fields of:

- Integrated sensing arrays [1];
- Stability of ratiometric optical thermometry [2];
- Near-infrared thermometry [3];
- Gas sensing and electrochemical properties of rare earth ferrite [4];
- Magnetic and humidity sensing properties of iron oxide nanoparticles [5];
- Magnetic ophthalmic realignment systems [6];
- Room temperature ammonia gas sensors [7];
- Using uniaxial polyvinylidene fluoride-based photoacoustic sensors [8];
- Dielectric properties of sodium and neodymium [9];
- Time-dependent demagnetization of magnets under magnetic fields [10];
- Effect of REO co-dopant on ionic conductivity [11];
- Synergy of neodymium and copper [12];
- Magnetically driven actuators for vector scanning mems mirrors [13];
- Neodymium-doped graphene foam for magnetic sensors [14];
- Non-contact fluorescence intensity ratio thermometer [15].

Basically, they are devoted to the use in ionic form or in the form of compounds such as perovskite or pyrochlore, but there are also studies related to synthesis and use in metal form [16,17]. An analysis of the market for rare earth metals (REM) consumption for the next decade and the dynamics of REM consumption in the past shows that the consumption of REM, and in particular neodymium, is constantly growing [18]. The undisputed leader in the production of REM and their compounds at the moment is China, which in turn is constantly raising prices for REM and their compounds. Starting from 2017, the price index in China for rare earth metals increased by 39.7%, and for compounds by 8.3% [17,18,19,20,21].

These price indices are calculated as the volume of exports of REM and REM compounds from China to all countries in monetary terms divided by the same indicator in physical terms [22,23,24,25]. Therefore, the solution of import substitution issues is also an urgent task. The aim of this work is to study and develop a technology for low-temperature synthesis of light-group halide REM compounds suitable for producing compact REM or ligatures based on them.

**Figure 1:**
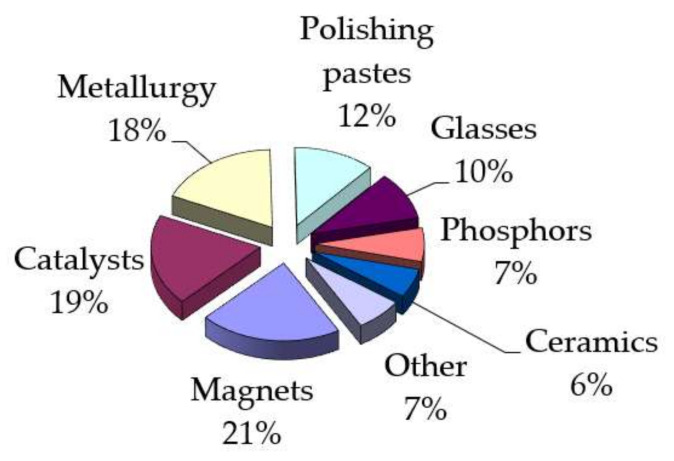
Structure of the global consumption of rare earth metals.

The relevance of the work is determined by the need to increase the production efficiency and improve the quality, with a constant (from 25 to 40% per year) growth in consumption, of individual rare earths in the form of metals or oxides in the most important industries (such as magnet production, nuclear industry, metallurgy, etc.). The structure of the global consumption of rare earth metals is presented in Figure 1.

Rare earth metals with magnetic properties (Pr, Nd, Sm, Tb, and Dy) are of particular interest, as they find application in the recently developed high-tech industries. These include electronics, space technology, electric vehicles, and green energy. Based on this, the authors in [18] made a forecast for the development of production and consumption of these metals in the near future (Table 1). According to numerous publications [19,20,21,22,23,24,25,26,27], the data show that there is a significant increase in the consumption of magnetic REM, and even an increase in production volumes will not fully meet the demand. This will undoubtedly affect the world prices of these metals. Therefore, the development of new technologies and methods of their production is an urgent task.

Neodymium, terbium, and dysprosium, which have increased magnetic properties compared to some ferromagnets, are the most popular magnetic materials among the entire range of REM. The Nd_2_Fe_14_B and Sm_2_Co_17_ magnets are now widely used in industries and entertainment. Methods of adding, in particular, dysprosium into magnetic alloys to increase the magnetic properties are proposed in [28,29]. However, obtaining these metals is associated with certain difficulties due to their high chemical activity. To obtain the entire range of REM, either electrolytic or metallothermic [30,31] methods can be used, and chlorides or fluorides can be used as starting materials. The obtained product is used in the production of neodymium-based alloys by a metallothermic reduction of a mixture of fluorides. The sensor material obtained in this way is characterized by a low (less than 0.05%) oxygen content. This work discusses a method for producing neodymium fluoride from its oxide for the production of the ligatures Nd-Fe.

## 2. Materials and Methods of Preliminary Analysis

**Figure 2:**
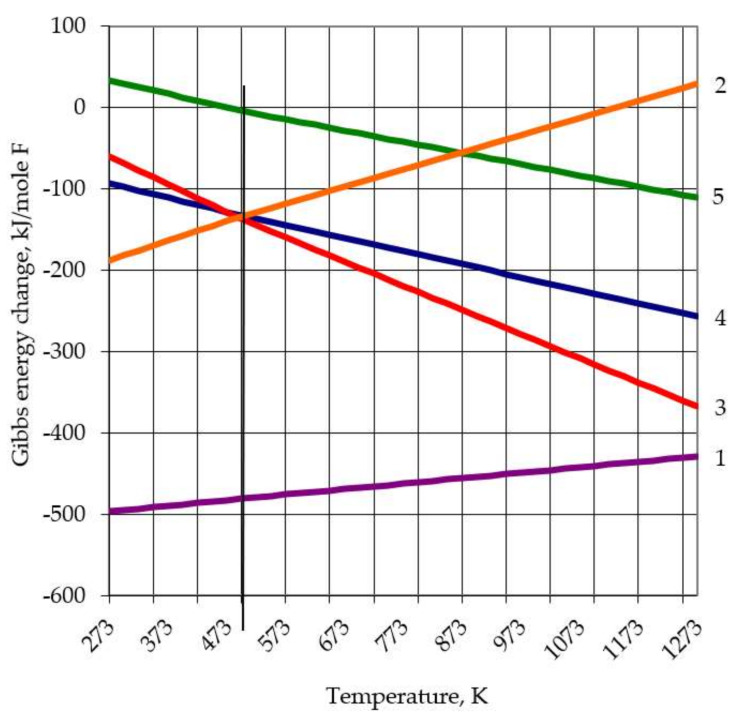
Gibbs energy change for neodymium oxide fluorination Equations (1)–(5).

REM fluorides can be obtained by fluorinating the oxides of these metals. Various fluorine-containing compounds or elemental fluorine are usually used as fluorinating reagents. Each of these reagents has its own advantages and disadvantages. For preliminary assessment and selection of the reagent, a thermodynamic analysis of Equations that can occur in the “neodymium oxide—fluorinating reagent” system was performed with the use of special software for thermodynamic equilibrium equations (ex. Thermo-Calc^tm^ or FACT-sage^tm^ online). The calculation results assigned to the mole of fluorine are shown in Figure 2.

1/3Nd_2_O_3_ + F_2_(g) = 2/3NdF_3_ + 1/2O_2_ (1)

1/3Nd_2_O_3_ + 2HF(g) = 2/3NdF_3_ + H_2_O(g) (2)

1/3Nd_2_O_3_ + 2NH_4_F(g) = 2/3NdF_3_ + 2NH_3_(g) + H_2_O(g) (3)

1/3Nd_2_O_3_ + NH_4_HF_2_(g) = 2/3NdF_3_ +NH_3_(g) + H_2_O(g) (4)

2NH_4_F = NH_4_HF + NH_3_(g) (5)

The most effective fluorinating agent is elemental fluorine. Its use is possible across a wide range of temperatures, but it also has certain disadvantages. Elemental fluorine is obtained by electrolysis of a mixture of KF and liquid HF. The melting point of this mixture is 70 °C. HF is produced by the action of sulfuric acid on fluorite (CaF_2_). This mineral is not widely distributed in nature, so elemental fluorine is an expensive reagent and is used for the production of the most valuable products, and where it cannot be replaced, for example, in the production of uranium hexafluoride. In addition, fluorine is one of the most aggressive reagents, which creates certain difficulties in choosing materials and the need to create safe working conditions and reduce environmental pressure on nature.

In [32,33], experimental data on the use of elementary fluorine are presented, where some of the above problems were successfully solved. Fluorination with anhydrous HF gas is possible up to a temperature of 1223 K. However, there are also problems with the durability of the equipment materials. It is necessary to use materials that are also resistant to hydrofluoric acid vapors at high temperatures. In addition, Equations (1) and (2) are reversible, so a large surplus of reagents is needed, which further increases the cost of the process, especially when using elementary fluorine. More widely used ammonium fluorides are fluoride and bifluoride [33]. Ammonium fluorides in the anhydrous state interact with many substances and form ammonium fluorometallates.

Crystal phases of various compositions were found in LnF_3_-NH_4_F systems [34]. NH_4_LnF_4_ and (NH_4_)3LnF_6_ were most reliably identified. Nd and Tb are characterized by the formation of tetrafluorometallates (NH_4_LnF_4_), which decompose directly to LnF_3_. The pressure in the temperature range 478…523 K for NH_4_NdF_4_ decomposition is approximated by the equation lg*Kp* = 18,523 − 10,201/*T*, and the enthalpy of decomposition is 195.3 kJ/mol [35]. Analyzing the graph in Figure 2, it can be seen that ammonium fluorides occupy an intermediate place between elementary fluorine and anhydrous hydrogen fluoride in terms of their fluorinating capacity. Reactions with elementary fluorine and anhydrous hydrogen fluoride are exothermic over the entire temperature range, and the probability of passing reactions decreases with increasing temperature. Reactions with ammonium fluorides due to the formation of ammonium fluorometallates take place at lower temperatures.

At higher temperatures, thermal decomposition of the fluorometallates occurs. This position is illustrated by the sequence of reactions by Baykov:

2NH_4_NdF_4_ = 2NdF_3_ + 2NH_4_F (6)

Nd_2_O_3_ + 4NH_4_HF_2_ = 2NH_4_NdF_4_ + 2NH_3_(g) + 3H_2_O(g) (7)

For the sequence (7)-(6)-(5) the total reaction is Equation (4).

Nd_2_O_3_ + 8NH_4_F = 2NH_4_NdF_4_ + 6NH_3_(g) + 3H_2_O(g) (8)

2NH_4_NdF_4_ = 2NdF_3_ + 2NH_4_F (9)

The total Equation for the sequence (8)-(9) is Equation (3). It is not possible to calculate the thermodynamics of the reaction sequences due to the incompleteness of the thermodynamic data base for most of the REM fluorometallates. The data calculated by the additive methods are not representative due to low accuracy. Ammonium fluoride at temperatures above 165–170 °C can only exist at an ammonia pressure greater than 101.3 kPa. The vertical line in Figure 2 crosses the Gibbs energy change lines for Equations (3)–(5) at a temperature of about 500 K. At this temperature, the ammonia vapor pressure for these reactions is 101.3 kPa. At temperatures of 353–453 K (80–180 °C), the oxides of all lanthanides are fluorinated with a significant exothermic effect. However, during the subsequent thermal decomposition of the ammonium fluorometallates, in addition to heat absorption due to their decomposition to simple fluorides, at temperatures above 165–170 °C (and especially above 235–240 °C), heat absorption occurs due to the highly endothermic decomposition processes of ammonium fluoride itself and the vaporization of ammonia and water. Thus, in general, Equations (3) and (4) are endothermic, and they require significant energy costs. In this case, the use of NH_4_HF_2_ is more preferable, since no heat will be expended on the dissociation of ammonium fluoride. The analysis of neodymium fluoride production processes has shown that the most acceptable fluorinating agent is ammonium hydrofluoride (NH_4_HF_2_), which was used in this work. The fluorination process begins at low temperatures. Therefore, the rate of temperature rise will be of great importance. The speed must be such that the solid-phase fluorination process has time to pass before the NH_4_HF_2_ transition to the gas phase and its decomposition take place.

The temperature can then be raised to the remove surplus of NH_4_HF_2_. As a result of the fluorination reaction, the reaction product will be NdF_3_ (at 100% fluorination) or a mixture of NdF_3_ + Nd_2_O_3_ (at incomplete fluorination, REM fluoride and the unreacted starting oxide will remain in the residue after fluorination, since NH_4_HF_2_ will leave the reaction zone when the temperature increases). The coefficient (*kF*) [35] was used to determine the degree of fluoridation during the experiment:

(10) $$k_{F}=\frac{m_{NdF_{3}}}{m_{prod}}$$

$m_{NdF_{3}}$—calculated mass of NdF_3_ (at 100 % fluorination), g;
$m_{prod}$—mass of the obtained product, g.

## 3. Principles and Results of Experimental Work

For experiments, neodymium oxide with a purity of 99.5% (2N) with average grain size less 0.8 µm was used, and dry ammonium hydrofluoride with a purity of at least 98.05%. The raw materials Nd_2_O_3_ and NH_4_HF_2_ powders were used as starting materials in a molar ratio of 1:8 (stoichiometric ratio for Equation (8)). The charges were weighted (2 ± 0.2 g) and filled in the crucibles. The crucibles were installed in the cassette, which was loaded into a horizontal tubular furnace with the ability to change the rate of heating and a programmed control with quartz retort. The heat temperature was raised from 25 up to 600 °C, with different heating rate of 3, 2, and 1 degree per minute. During and after the process, the crucibles were sequentially extracted using the calculated time intervals after heating. The determination of the neodymium fluoride yield according to the coefficient (10) was made. At the end of the experiment and the preliminary express evaluation, the sample was sent for X-ray phase analysis.

The wave X-ray fluorescence spectrometer ARL 9900 WS was used for the analysis. Elemental analysis and phase analysis of the sample were performed.

For elemental analysis, the sample was placed in a metal holder with a plastic centering ring. The samples were analyzed in a vacuum. The standardless analysis was carried out according to the program developed for the software Uniquant. The standard Kappa list “AnySample” was used for the calculation, and the calculation was carried out under the assumption of an oxide matrix. The total analysis time of one sample was 18 min, while measurement takes place on 126 analytical lines. Qualitative phase analysis was performed using the ICDD PDF-2 database and the CrystallographicaSearch-Match software package.

On the basis of the obtained data, kinetic graphs of the process were constructed.

**Figure 3:**
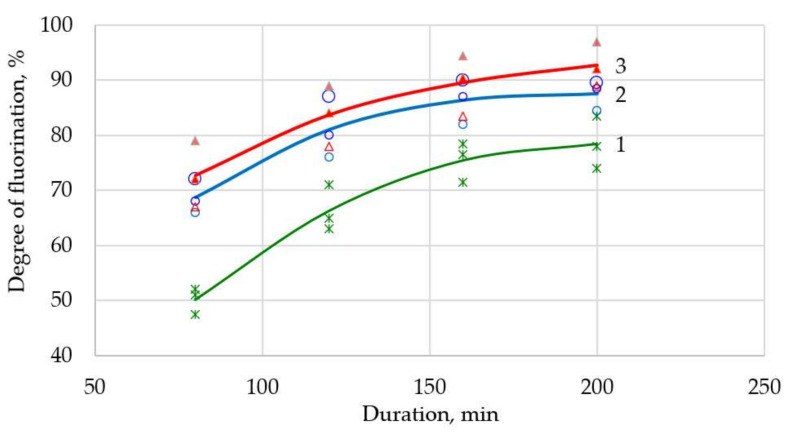
Neodymium fluoride yield as a function of the fluorination time at a heating rate of 3 degrees per minute up to 600 °C (surplus NH_4_HF_2_: 1—0%; 2—10%; 3—20%).

**Figure 4:**
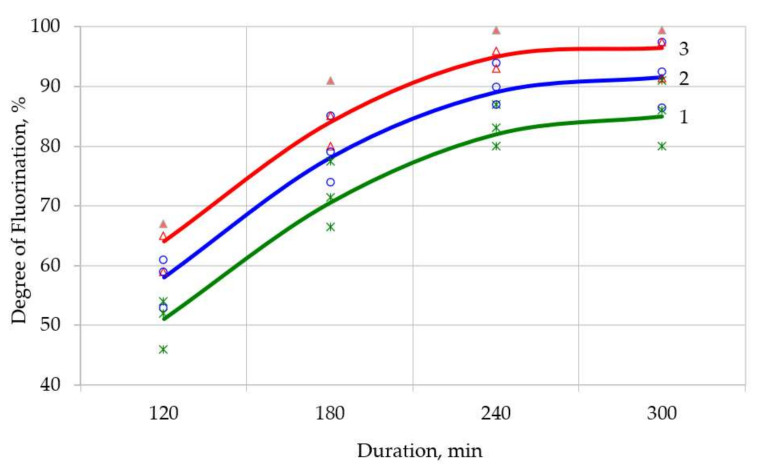
Neodymium fluoride yield as a function of the fluorination time at a heating rate of 2 degrees per minute up to 600 °C (surplus NH_4_HF_2_: 1—0%; 2—10%; 3—20%).

**Figure 5:**
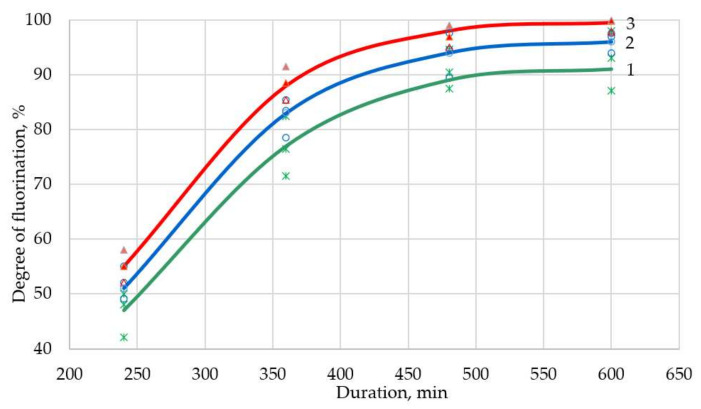
Neodymium fluoride yield depending on the fluorination time at a heating rate of 1 degree per minute up to 600 °C (surplus NH_4_HF_2_: 1—0%; 2—10%; 3—20%).

**Figure 6:**
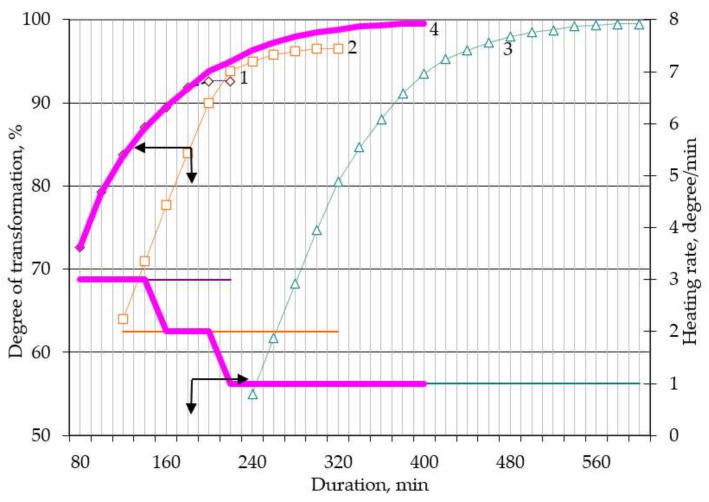
Processed total graph of theoretical yield under variable heating at different stages, surplus NH_4_HF_2_ 10%. (neodymium fluoride yield depending on the fluorination time at a heating rate of degrees per minute: 1—3; 2—2; 3—1; 4—total graph).

In order to increase productivity and the degree of chemical transformation, it was proposed to perform heating stepwise; i.e., at the initial stage, heat at a speed of 3 degrees per minute, after which the heating speed was reduced to 2 degrees per minute, and at the final stage, the speed was reduced to 1 degree per minute. Heating modes were identified in Figure 3, Figure 4 and Figure 5. Using the least squares method, these modes were superimposed on each other to obtain a correlation coefficient of 96%. The theoretical extraction plot obtained by this iteration is shown in Figure 6.

A series of experiments were conducted to evaluate the correctness of the assumptions. After careful mixing of the components, the suspension in the form of powder was weighed, placed in an aluminum ceramic boat, which in turn was placed in a quartz retort in a tube furnace (Carbolite MTF 12/25/250). The charge heating mode was set according to the specified program (Table 2). During the process, the charging boat was removed, weighed, and placed back in the oven at set intervals. Based on the developed express analysis, the degree of transformation was determined. Similar actions were performed after the end of the process. The results of the experiments are shown in Figure 3, Figure 4 and Figure 5.

With a different surplus of ammonium hydrofluoride, the yield of fluorination process does not change after 3 h. At this (3 degrees per minute) rate of heating, the solid-phase fluorination reaction does not have time to pass, since part of the fluorinating reagent has time to volatilize or decompose. Surplus of NH_4_HF_2_ leads to a higher degree of fluorination.

Carrying out fluoridation at a rate of temperature rise of 2 and 1 degrees per minute, although it makes the process longer, increases the degree of fluoridation significantly (Figure 4 and Figure 5), reaching almost 100%. Increasing the process time makes it possible to pass the solid-phase section of fluorination. The temperature of 500–600 °C allows to completely remove the surplus of hydrofluoride from the reaction zone and get a solid product of neodymium fluoride.

Thermodynamic calculations in the preliminary section and analysis of the kinetic graphs show that ammonium hydrofluoride is an acceptable fluorinating agent.

**Figure 7:**
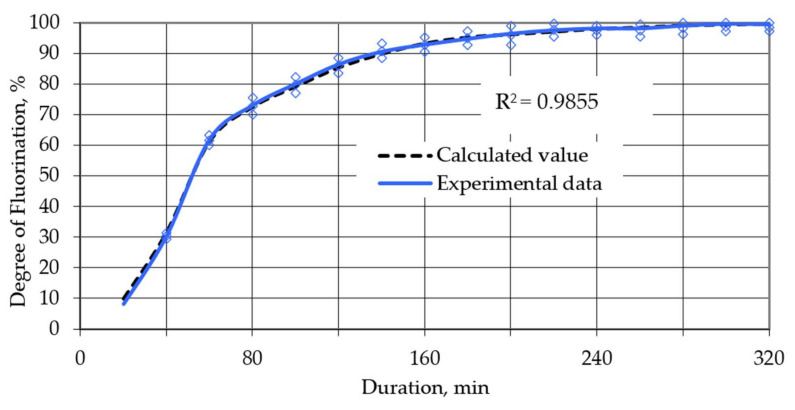
Dependence of the degree of transformation on time for a given heating mode.

The total graph (Figure 6) was obtained as summarized graphs with a different heating rate and smoothed transitions among them. The data were processed using the least squares method by adding intersecting points. The results of processing the received data are shown in Figure 7. So, we obtained the sequence of the heating rate change (Table 2).

## 4. Discussion of Results

**Figure 8:**
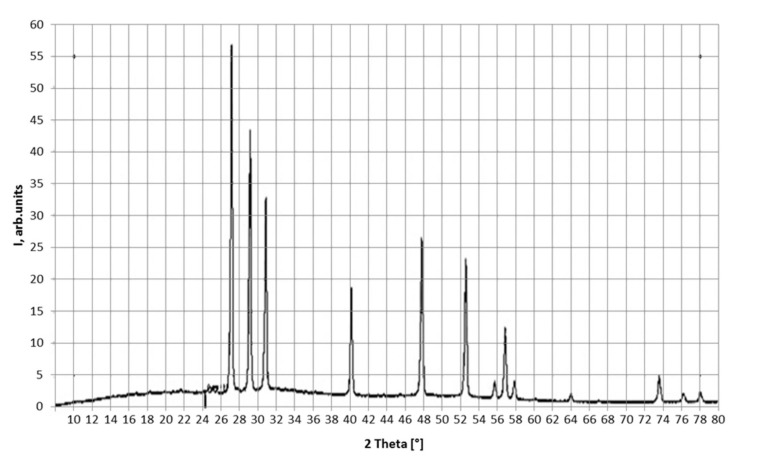
Diffractogram of the NdF_3_ sample.

The thermodynamic possibility of obtaining neodymium fluoride using the process of solid-phase low-temperature fluorination was given. The optimal kinetic parameters of the technological process, such as an excess of 10% ammonium fluoride and a heating rate of 1 °C/min to 600 °C, were determined. An excess of ammonium fluoride leads to an increase in the degree of fluorination (a 20% ammonium fluoride surplus leads to the process to be carried out faster by an average of 10%; however, the process of removing the excess fluorinating agent from the product leads to additional energy and time costs, which worsens the efficiency of the process). Carrying out fluorination at a heating rate of 1 °C/min increases the duration of the process, but at the same time it increases the degree of fluorination. An increase in the duration of the process makes it possible to pass the reaction in the solid-phase region. Carrying out the process at a temperature of 600 °C allows to completely remove the excess ammonium fluoride from the reaction zone, completely convert the intermediate phase of NH_4_NdF_4_ into NdF_3_, and thus obtain anhydrous NdF_3_. Figure 8 shows a diffractogram of the NdF_3_ sample.

Table 3 shows the phase composition of the neodymium oxide samples.

Based on a comparison of existing methods, it can be said that the fluorination process using ammonium fluoride as a fluorinating reagent has economic advantages in comparison to methods with gaseous fluorine and hydrogen fluoride reagents, since there is no need to use expensive gaseous fluorine and a large amount (250% of the stoichiometric required amount) of hydrogen fluoride. In addition to the fact that the method turned out to be very cost effective, the method is safer, compared to others, to the environment.

The method of variable heating at different stages (in accordance to reduce the time for the process) was also tested. Analysis of the results shows that the degree of correlation between the theoretical and experimental curves is 98.55%, which indicates that our assumptions are correct. According to the obtained kinetic curve, the activation energy (8461.7 kJ/mol) was determined, and the change in the limiting stage from vaporization to internal diffusion was also established. Thus, based on the results of research on optimizing low-temperature fluorination, the following technological parameters for obtaining NdF_3_ can be proposed:

- Surplus of NH_4_HF_2_ 10%;
- Heating rate up to 600 °C sequentially reduced, (3, 2, 1) °C/min;
- Total process duration 320–360 min.

Due to proposed heating mode the same productivity and the yield of chemical transformation were achieved, with an increased efficiency up to 30%, which can significantly reduce the cost of production, providing the necessary indicators for competing in the market of REM-based magnetic sensors for geotechnical surveys.

## 5. Conclusions and Final Remarks

A projected increase in the demand for magnetic REM will stimulate research investigations in the field of their production.

Thermodynamic analyses of the “Nd_2_O_3_-fluorinating agent” system, showed that using ammonium hydrofluoride in comparison with F_2_ and HF allows the process to be carried out at lower temperatures.

Using the previously developed express analysis, kinetic studies of the process of fluorination of neodymium oxide with the production of fluoride suitable for the production of metal were carried out.

The following technological parameters for obtaining neodymium fluoride were established:

- Surplus of ammonium hydrofluoride: 10% relative to the stoichiometric amount;
- Heating rate up to 600 °C: sequentially reduced from 3 to 1 °C/min;
- Degree of fluorination: 99.0–99.8%

The continuously growing demand for magnetic materials used in geotechnical, optical, and medical sensors leads to the need to find ways to reduce the costs of producing metals and ligatures based on neodymium. One of these methods is low-temperature fluorination, which allows avoiding the use of expensive reagents, reducing the temperature of the process and reducing its duration. Using the least squares method we obtained the output curve of the process, with a high level of confidence, and the correlation coefficient, which definitely indicates the correctness of the chosen methodology and the prospects of the process.

## Figures and Tables

**Table 1:** Balance of magnetic REM world production and consumption in 2020 and for the 2030 forecast.

Metal	2020			2030		
	Production	Consumption	Balance	Production	Consumption	Balance
Pr	10.3 *	15.1	−4.8	14.6	24.6	−10.0
Nd	32.9	40.8	−7.9	47.1	64.2	−17.1
Sm	3.5	1.2	2.3	5.2	2.0	3.2
Tb	0.5	0.4	0.1	0.7	0.6	0.1
Dy	2.3	3.7	−1.4	3.5	8.1	−4.6
Total	49.5	61.2	−11.7	71.1	99.5	−28.4

* Values in the table in thousand tons.

**Table 2:** The heating mode regime.

Stage	Duration, Minutes	Temperature of Reducing Rate, °C	Heating Rate, Degree per Minute
1	120–140	380	3
2	20–30	420	2
3	180–220	600	1

**Table 3:** Phase composition of the NdF_3_ sample.

Phase	Composition	Content, % (by Weight)
Neodymium Fluoride	NdF_3_	100

## Data Availability

Data is contained within the article.
